# Supplementary material for: A comprehensive single-cell map of T cell exhaustion-associated immune environments in human breast cancer
Source: Nat Commun. 2023 Jan 6;14:98. doi: 10.1038/s41467-022-35238-w (PMC9822999; doi:10.1038/s41467-022-35238-w)
Supplement: Supplementary file 1 — Supplementary Information [file 41467_2022_35238_MOESM1_ESM.pdf]

Supplementary Fig. 1

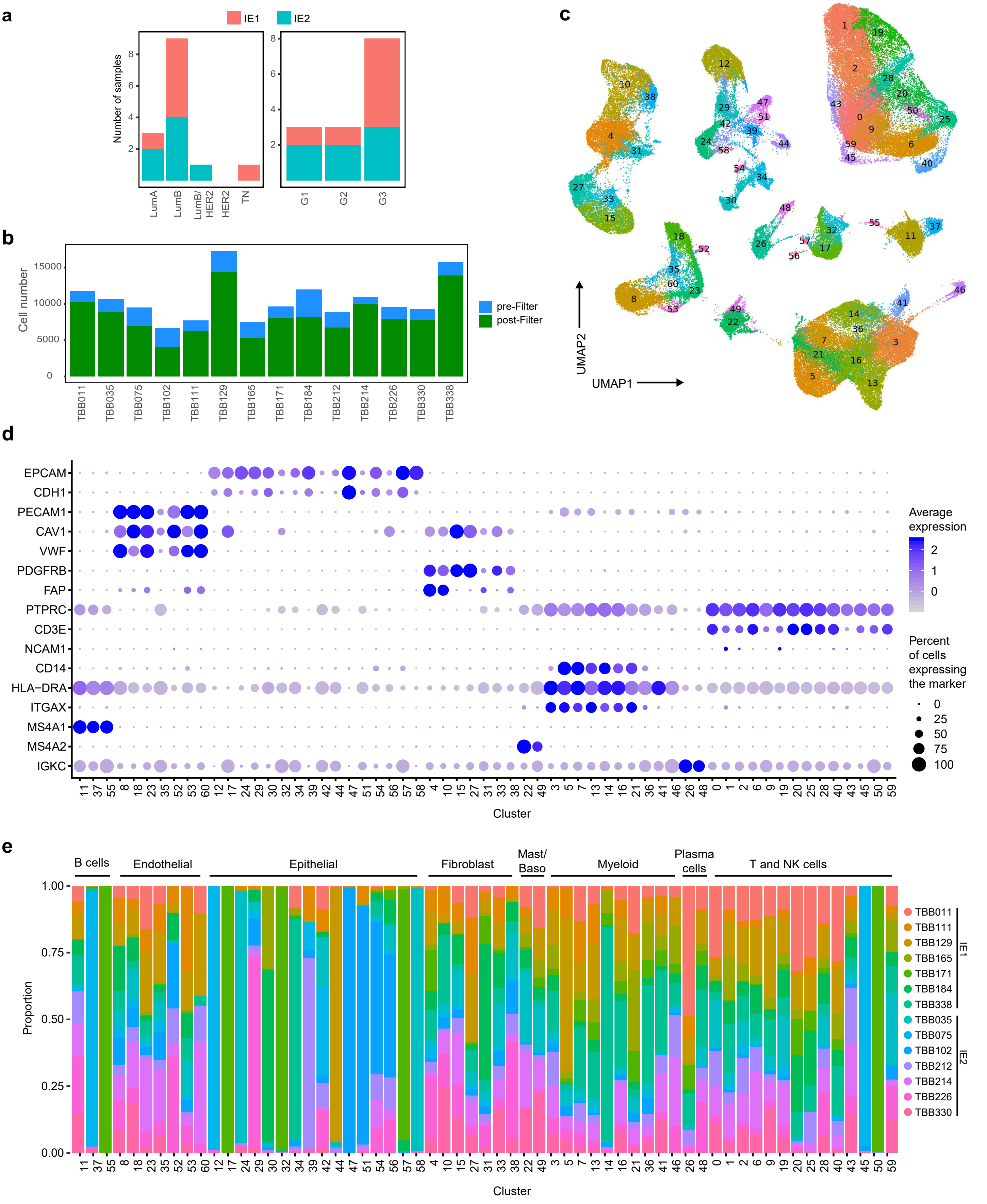

Supplementary Fig. 1: A single-cell transcriptomic atlas of exhausted and non-exhausted breast tumor microenvironments. **(a)** Number of patient samples subjected to sequencing by tumor subtype (left) and tumor grade (right). **(b)** Number of sequenced cells per patient sample before and after cell filtering. **(c)** UMAP plot of scRNA-seq data colored by cluster from initial high-resolution Seurat clustering. **(d)** DotPlot of scRNA-seq data showing expression of indicated markers in the high-resolution clusters. **(e)**Stacked barplots showing the proportion of cells originating from each patient sample in each of the full-dataset clusters (scRNA-seq data).

Supplementary Fig. 2

a

Backbone panel

Antigen	Description
Histone H3	Nuclear marker
SMA	Tissue structure
Ki-67	Proliferation
HLA-ABC	MHC-I
CD31 & vWF	Endothelial cells
pan-Cytokeratin	Epithelial cells
E-Cadherin	Epithelial cells
CD3	T cells
CD4	TCR co-receptor
CD8	TCR co-receptor
CD7	T & NK cells
Myeloperoxidase	Neutrophils
HLA-DR	MHC-II
CD68	Macrophages
CD11c	Myeloid cells
LAMP3	Activated DCs
CD15	Granulocyte
PD-L1	PD-1 ligand

Protein panel: Additional markers

Antigen	Description
CD140b	Fibroblast subset
CA IX	Hypoxia
Cleaved Caspase & cleaved PARP	Apoptosis
CD38	Ectoenzyme
CD27	T cell activation
PD-1	Co-inhibitory R.
GITR	Co-stimulatory R.
LAG-3	Co-inhibitory R.
TCF1/TCF7	Transcription F.
FOXP3	Regulatory T cells
ICOS	Co-stimulatory R.
Tim-3	Co-inhibitory R.
CD20	B cells
CD21	Complement R.
CD40	APC activation
CD45RO	Pan immune
CD45RA	Pan lymphocyte
CD303	Plasmacytoid DC
IDO1	Immunoreg. enzyme
iNOS	Arginine metabolism
Arginase 1	Arginine metabolism
MMP9	Matrix metalloprotease
Granzyme B	Serine protease

RNA panel: Additional markers

Antigen	Description
CD11b	Complement R. 3
CD204	Scavenger R.
CD163	Scavenger R.
CD16	FCGR3a
CD14	LPS co-R.
CLEC9A	Lectin-like R.
CD1c	Lipid Ag Present.
CXCL13	Chemokine

Transcript	Description
CSF1	Growth Factor
CCL2	Chemokine
CCL4	Chemokine
CCL5	Chemokine
CCL17	Chemokine
CCL18	Chemokine
CCL22	Chemokine
CXCL9	Chemokine
CXCL10	Chemokine
CXCL13	Chemokine
MS4A1	B cells (CD20)
DapB	negative control

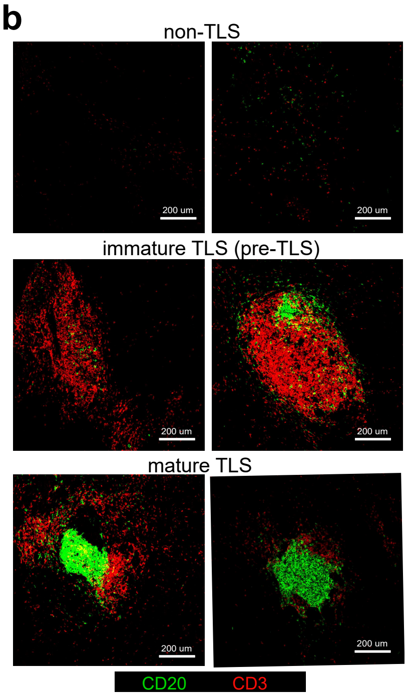

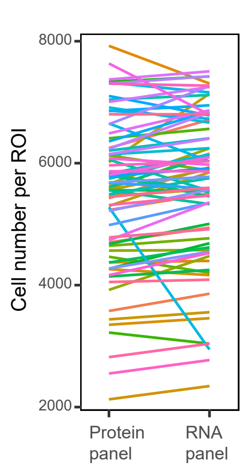

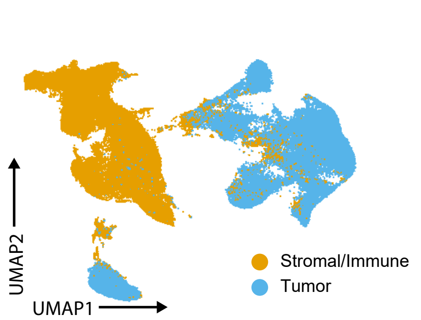

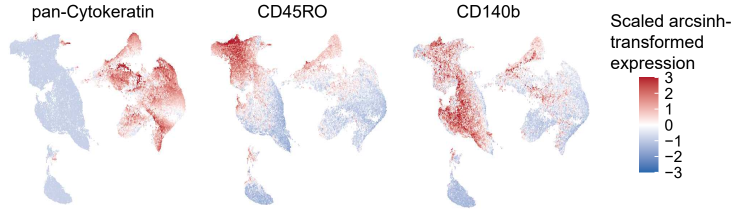

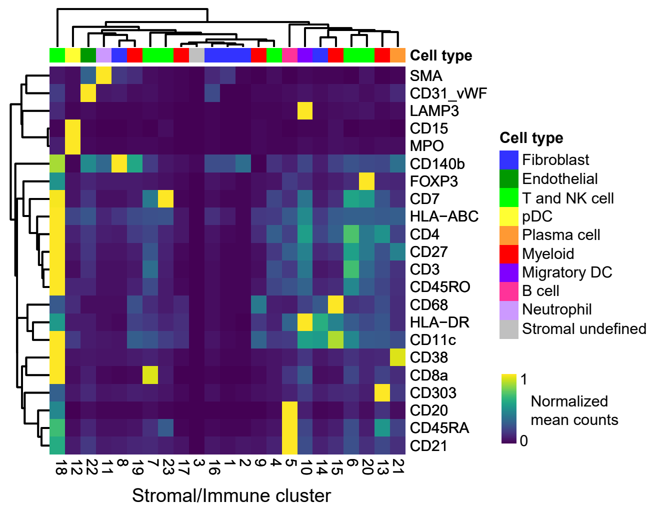

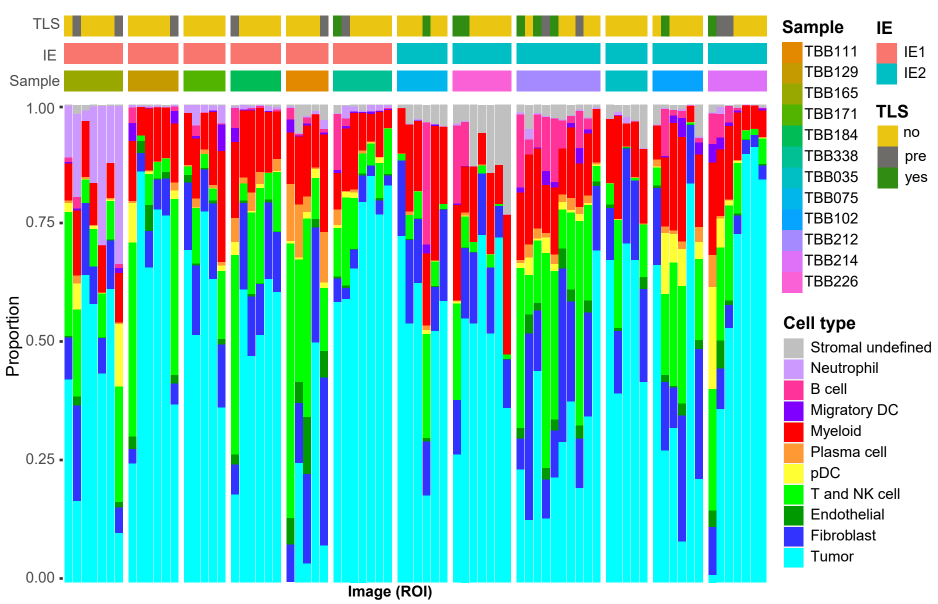

Supplementary Fig. 2: IMC setup and cell type annotation based on IMC Protein Panel data. (a) List of antigens and transcripts detected using both panels (left), only the Protein Panel (middle), or only the RNA Panel (right). **(b)** Exemplary IMC images of regions classified as non-TLS, immature TLS (pre-TLS), or mature TLS. The selected images are representative of cellular patterns seen across the dataset (n total = 77 images).**(c)** Line plot comparing total cell numbers in matched images from the Protein Panel and the RNA Panel datasets. **(d)** UMAP plot of IMC Protein Panel data of a random subset of 10,000 cells colored by cell class. **(e)** UMAP plots of IMC Protein Panel data colored by marker expression. **(f)** Heatmap of IMC Protein Panel data showing normalized average expression of selected marker genes for all stromal/immune subclusters. **(g)** Stacked barplot of IMC protein panel data displaying cell type compositions for all individual images (annotated with information about patient sample, immune environment (IE) classifica-tion and TLS status).

Supplementary Fig. 3

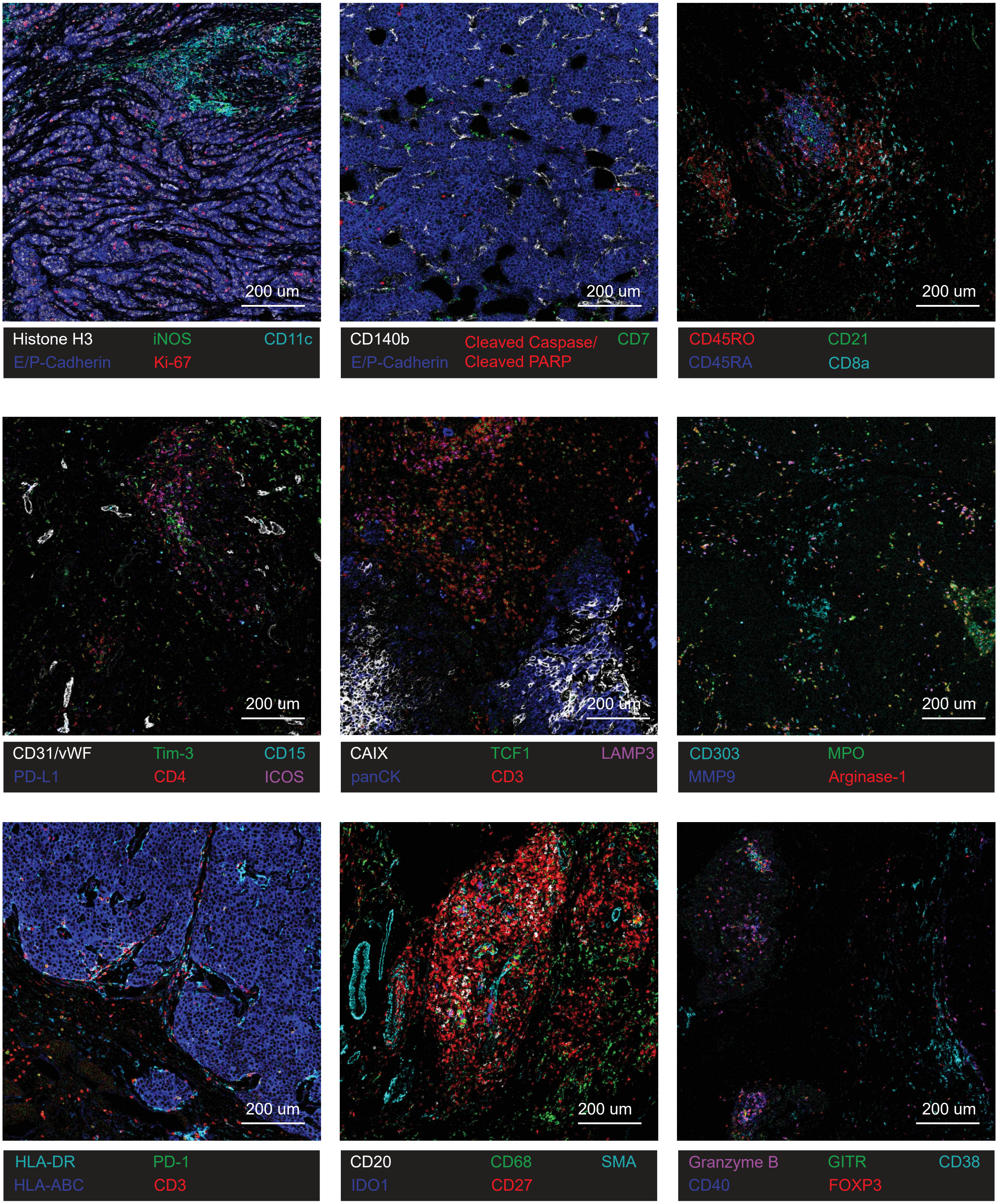

Supplementary Fig. 3: Staining patterns of all antibodies included in the Protein Panel on exemplary IMC images of breast cancer samples from this cohort. The selected images are representative of staining patterns seen across the dataset (n total = 77 images).

Supplementary Fig. 4

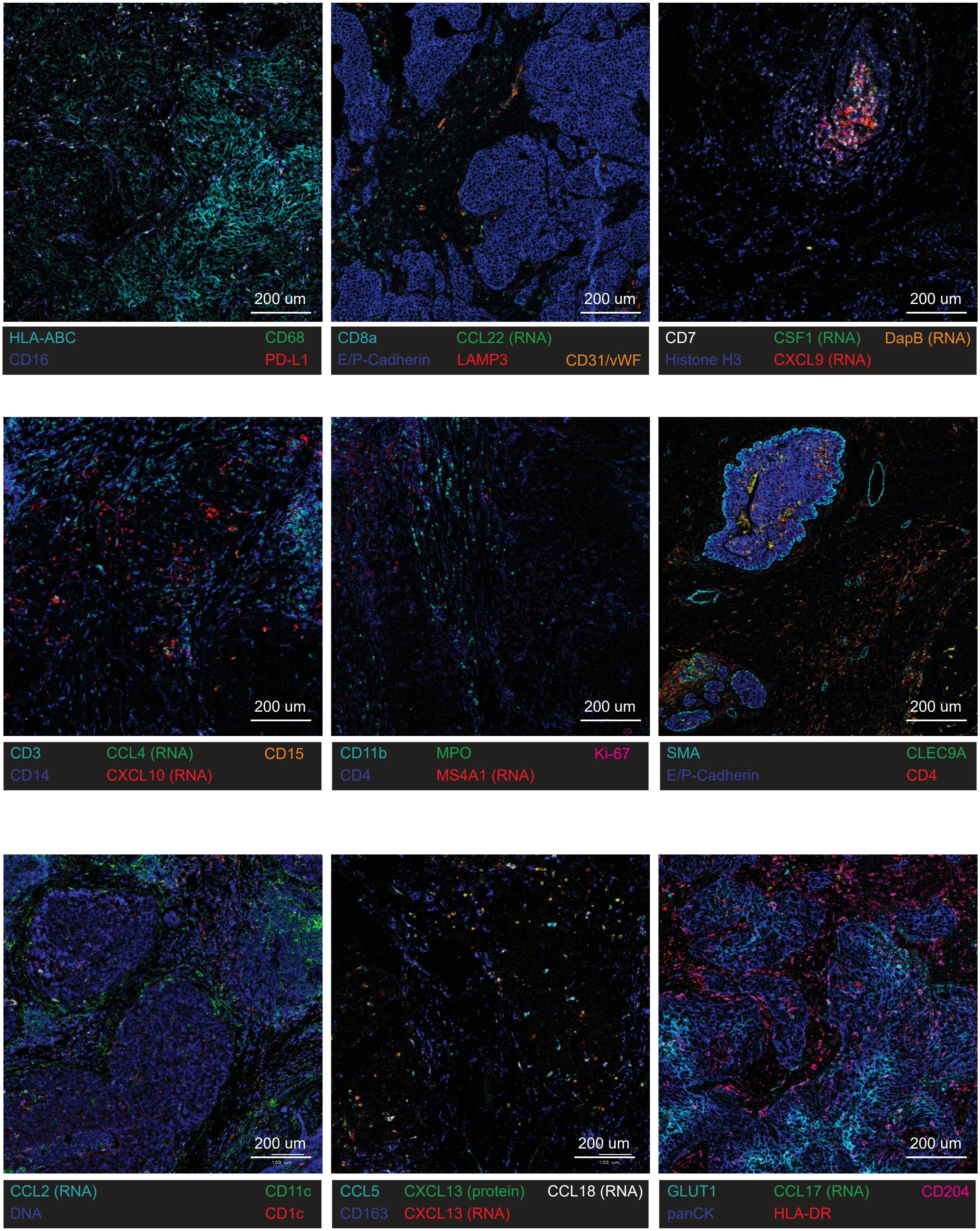

Supplementary Fig. 4: Staining patterns of all antibodies included in the RNA Panel on exemplary IMC images of breast cancer samples from this cohort. The selected images are representative of staining patterns seen across the dataset (n total = 77 images).

Supplementary Fig. 5

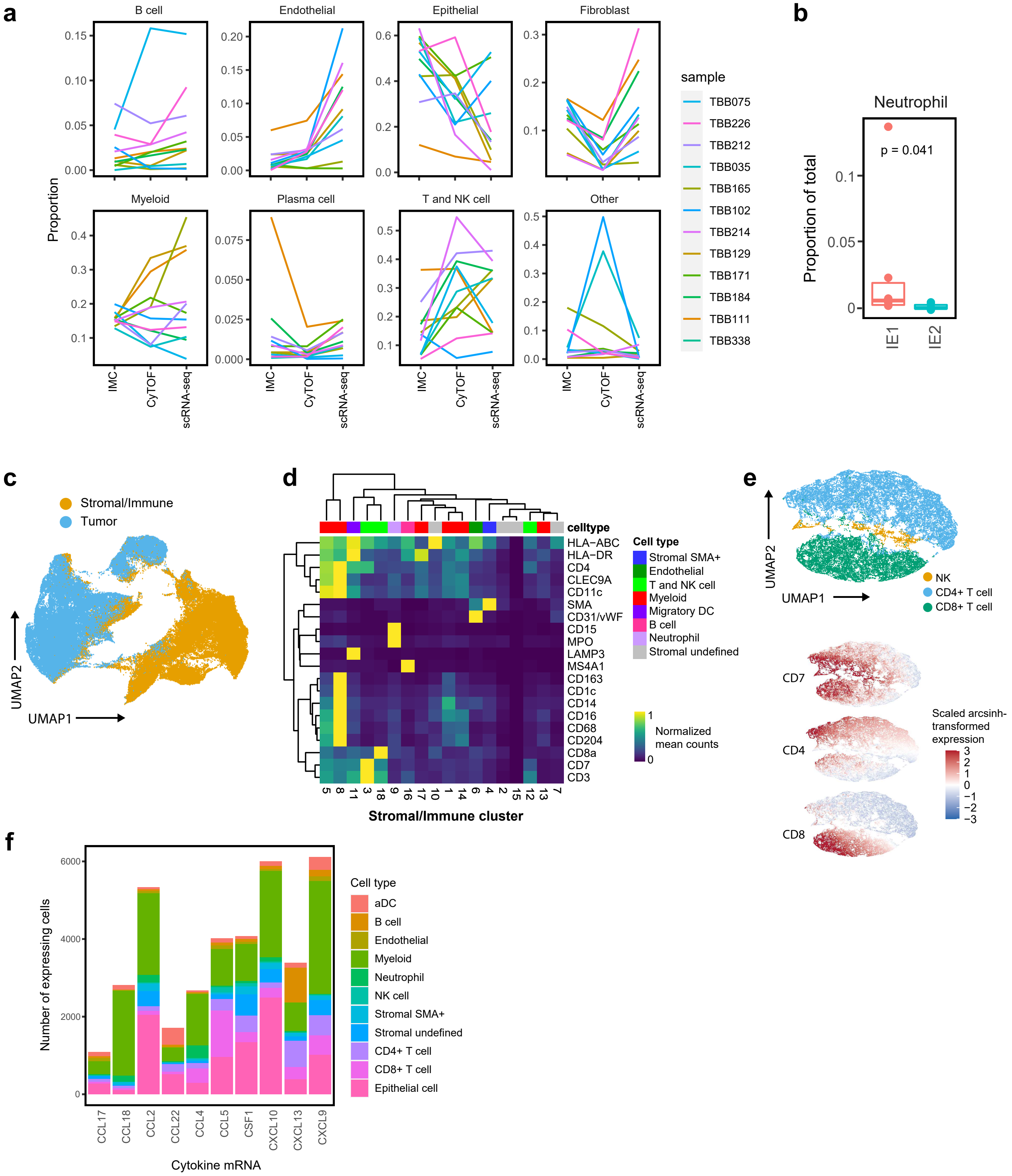

Supplementary Fig. 5: Cell type annotation and cytokine expression definition based on IMC RNA Panel data. (a) Line plot comparing relative cell type frequencies as determined by IMC, CyTOF and scRNA-seq analysis of the same sample. Only samples analyzed with all three technologies are included. **(b)** Neutrophil frequency in IE1 and IE2 tumors (IMC Protein Panel data, n = 12 independent patient samples). A two-sided paired Wilcoxon rank sum test was used for statistical analysis. Boxplot centers indicate the group median, boxplot bodies show IQR, and whiskers extend to the largest and the smallest value lying within 1.5 times the IQR above the 75th percentile and below the 25th percentile, respectively. **(c)** UMAP plot of IMC RNA Panel data of a random subset of 10,000 cells colored by cell class. **(d)** Heatmap of IMC RNA panel data showing normalized average expression of selected marker genes for all stromal/im-mune subclusters. **(e)** UMAP plots IMC RNA Panel data of all T and NK cells colored by subtype (top) and marker expression (bottom). **(f)** Stacked barplot showing the frequency of positive cells for each of the measured cytokine mRNAs, colored by cell type.

Supplementary Fig. 6

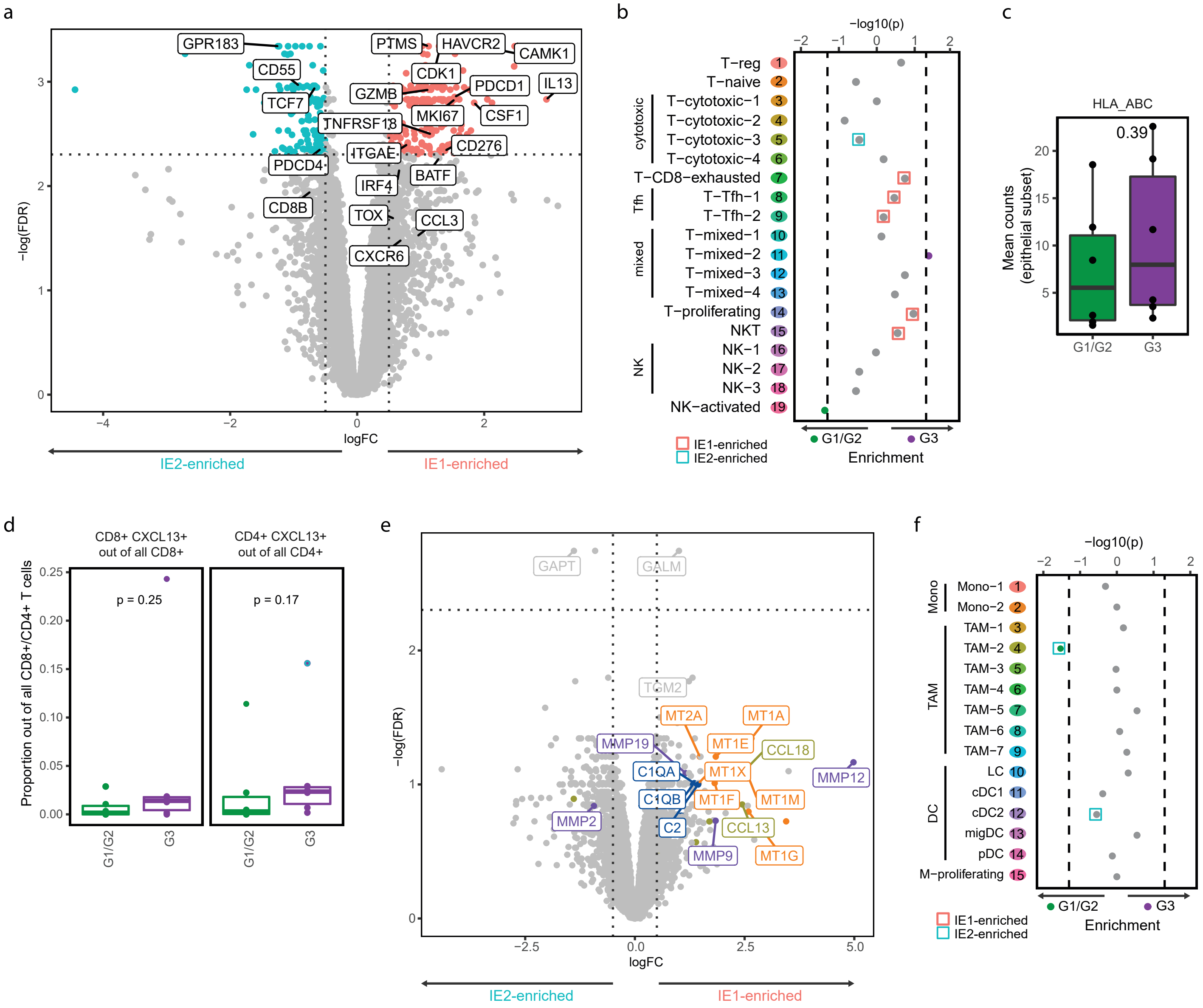

Supplementary Fig. 6: Tumor grade does not account for the observed differences between exhausted and non-exhausted immune environ-ments. (a) Volcano plot showing differential gene expression between T and NK cells of IE1 and IE2 samples in pseudobulk patient-av-eraged scRNA-seq data when tumor grade is used as a blocking factor in the model (compare Fig. 2A). Dashed lines indicate a false discovery rate (FDR) of 0.1 and a log2 fold change (logFC) of 0.5. **(b)** Enrichment of T/NK cluster frequencies, annotated by cell type, in lower grade (G1/G2) or higher grade (G3) samples. Wilcoxon rank sum test was used for statistical analysis, and dashed lines indicate a p = value of 0.05. Enrichment of the respective cluster in IE1 or IE2 is indicated by a colored box (compare Fig. 2E). **(c)** Boxplot comparing mean single-cell HLA-ABC expression in IMC data for the epithelial subsets of lower grade (G1/G2) versus higher grade samples (G3) (compare Fig. 2I). n = 14 independent patient samples. **(d)** Boxplots comparing CXCL13high cell proportions out of all CD8+ T cells (left) and CD4+ T cells (right) between IMC samples of lower grade (G1/G2) versus higher grade (G3) (compare Fig. 2K). Only non-TLS images were included. n = 14 independent patient samples. **(e)** Volcano plot showing differential gene expression between myeloid cells of IE1 and IE2 samples in pseudobulk patient-averaged scRNA-seq data when tumor grade is used as a blocking factor in the model (compare Fig. 4A). Dashed lines indicate an FDR of 0.1 and a logFC of 0.5. Genes are colored by function-al group. **(f)** Enrichment of myeloid cluster frequencies in lower grade (G1/G2) or higher grade samples (G3). Wilcoxon rank sum test was used for statistical analysis, and dashed lines indicate a p = value of 0.05. Enrichment of the respective cluster in IE1 or IE2 is indicated by a colored box (compare Fig. 4C).

For boxplots, two-sided wilcoxon rank sum test was used for statistical analysis. Boxplot centers indicate the group median, bodies show IQR, and whiskers extend to the largest and the smallest value lying within 1.5 times the IQR above the 75th percentile and below the 25th percentile, respectively.

Supplementary Fig. 7

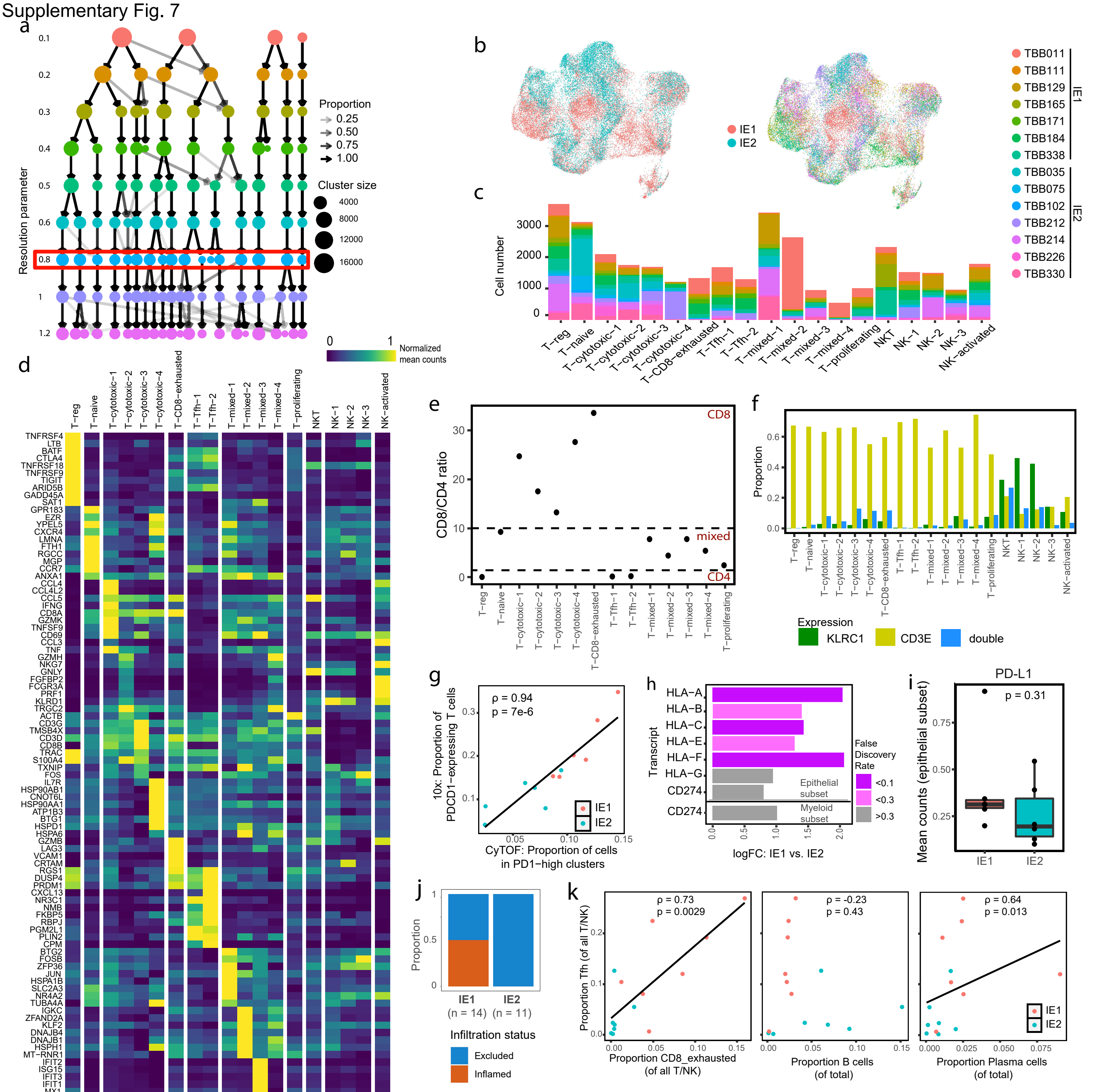

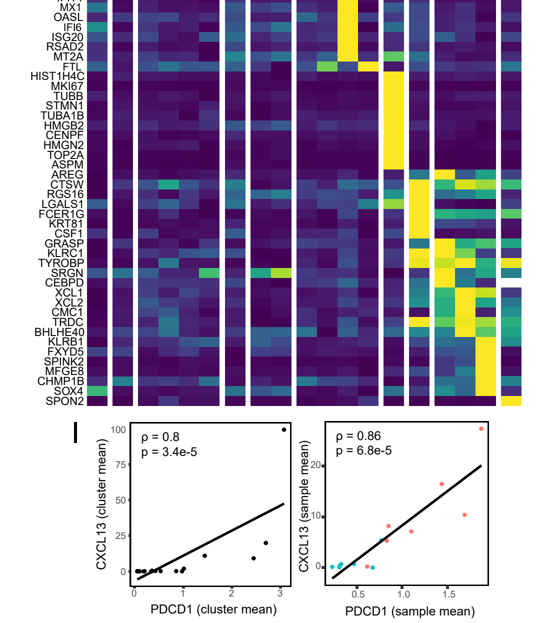

Supplementary Fig. 7: Detailed transcriptomics-based T and NK cell subcluster analysis. (a) Clustering of T and NK cell scRNA-Seq data with different clustering parameters. **(b)** UMAP plot of scRNA-seq data of 36,000 T and NK cell cells colored by IE (left) and patient sample (right). **(c)** Stacked barplots of the absolute cell number originating from each patient sample in each T and NK cell cluster (scRNA-seq data). **(d)** Heatmap of normalized average expression of the top 10 differentially expressed genes of each T and NK cell cluster (see also Supplementary Data 2A). **(e)** Ratio of mean CD8 and mean CD4 expression for each T cell cluster. To obtain mean CD8 expression, mean CD8A and mean CD8B expression were averaged. Dashed line indicates where cutoff for CD8+ T cell clusters was made. **(f)** Bar chart indicating proportion of cells that express either KLRC1 or CD3E or both for each T and NK cell cluster. **(g)** Scatterplot of the frequency of PDCD1-expressing T and NK cells in scRNA-seq data versus the frequency of T cells belonging to PD-1high clusters in CyTOF data for all patient samples. **(h)** Bar plot showing the log2-fold expression change of MHC-I transcripts and CD274 in the epithelial subset and CD274 in the myeloid subset from IE1 relative to IE2 (patient-averaged pseudobulk data). **(i)** Boxplot of IMC protein panel data comparing the mean PD-L1 protein expression for the epithelial subset of IE1 versus IE2 samples (n total = 12 biologically independent patient samples). Two-sided wilcoxon rank sum test was used for statistical analysis. Boxplot centers indicate the group median, boxplot bodies show IQR, and whiskers extend to the largest and the smallest value lying within 1.5 times the IQR above the 75th percentile and below the 25th percentile, respectively. **(j)** Stacked barplots indicating the slide-wide CD8+ T cell infiltration status for a cohort of 14 IE1 samples and 11 IE2 samples (including the samples analyzed by IMC). **(k)** Scatterplot of Tfh frequency versus the frequency of CD8+ exhausted T cells (left), and B cells (middleright) and plasma cells (right) as defined by scRNA-seq. **(l)** Scatterplot of mean CXCL13 transcript abundance versus mean PDCD1 transcript abundance for each T and NK cell cluster (top) and each patient sample (bottom).
For scatterplots, Spearman correlation coefficient (two-tailed test) and p value are indicated.

Supplementary Fig. 8

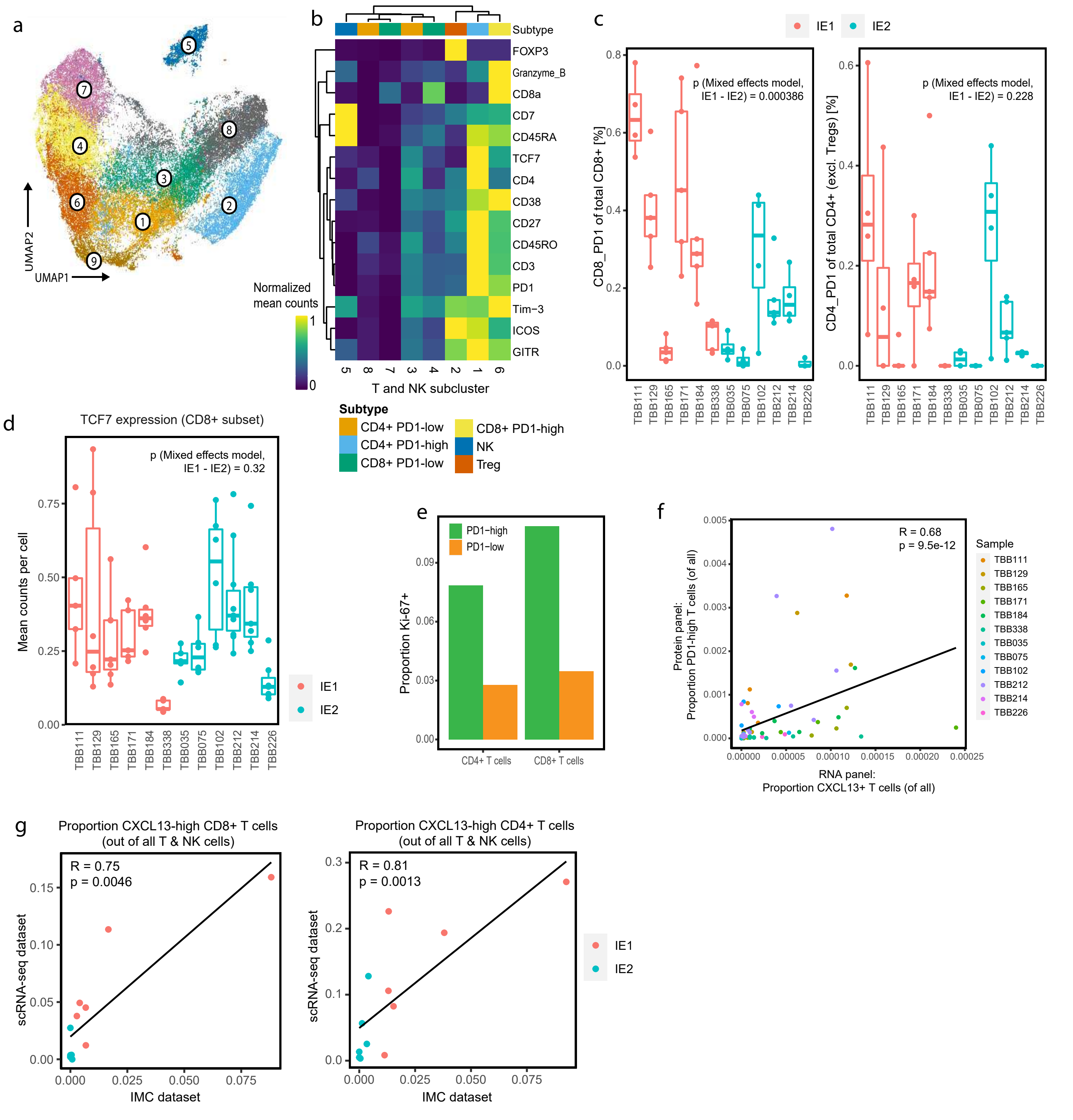

Supplementary Fig. 8: IMC-based T cell subclustering and comparison to scRNA-seq. (a) UMAP plot of all T and NK cells colored by subcluster based on IMC data (Protein Panel). Cluster 9 was excluded from further T cell subtype analysis as it was high for all markers and present in only one sample. **(b)** Heatmap of normalized average expression of the most relevant markers for each T and NK cell subcluster in IMC data (Protein Panel). Subtype annotation is displayed above the heatmap. **(c)** Boxplot comparing PD-1high cell proportions out of all CD8+ T cells (left) and CD4+ T cells (right) between IE1 and IE2 samples based on IMC data (Protein Panel). Each dot represents one image and only non-TLS images were included (n total = 53 images). Mixed effects models were fitted on the sqrt-transformed data. **(d)** Boxplot comparing the mean TCF7 expression between CD8+ T cells from IE1 and IE2 samples based on IMC data (Protein Panel). Each dot represents one image (n total = 77 images). A mixed effects model was fitted on the sqrt-transformed data. **(e)** Barplots showing the proportion of Ki-67+ cells for PD-1high and PD-1low T cell subsets based on IMC data (Protein Panel). **(f)** Scatterplot of the CXCL13+ T cell proportion versus the PD-1high T cell proportion for matched ROIs from the RNA Panel and the Protein Panel, respec-tively. **(g)** Scatterplot of CXCL13high cell proportions as defined by IMC versus scRNA-seq for CD8+ T cells (left) and CD4+ T cells (right). A significant correlation persists even after removal of the apparent outlier.

For scatterplots, Spearman correlation coefficient (two-tailed test) and p value are indicated. Boxplot centers indicate the group median, boxplot bodies show IQR, and whiskers extend to the largest and the smallest value lying within 1.5 times the IQR above the 75th percentile and below the 25th percentile, respectively. For panels C and G, only non-TLS images were included in the analysis in order to avoid region-selection bias.

Supplementary Fig. 9

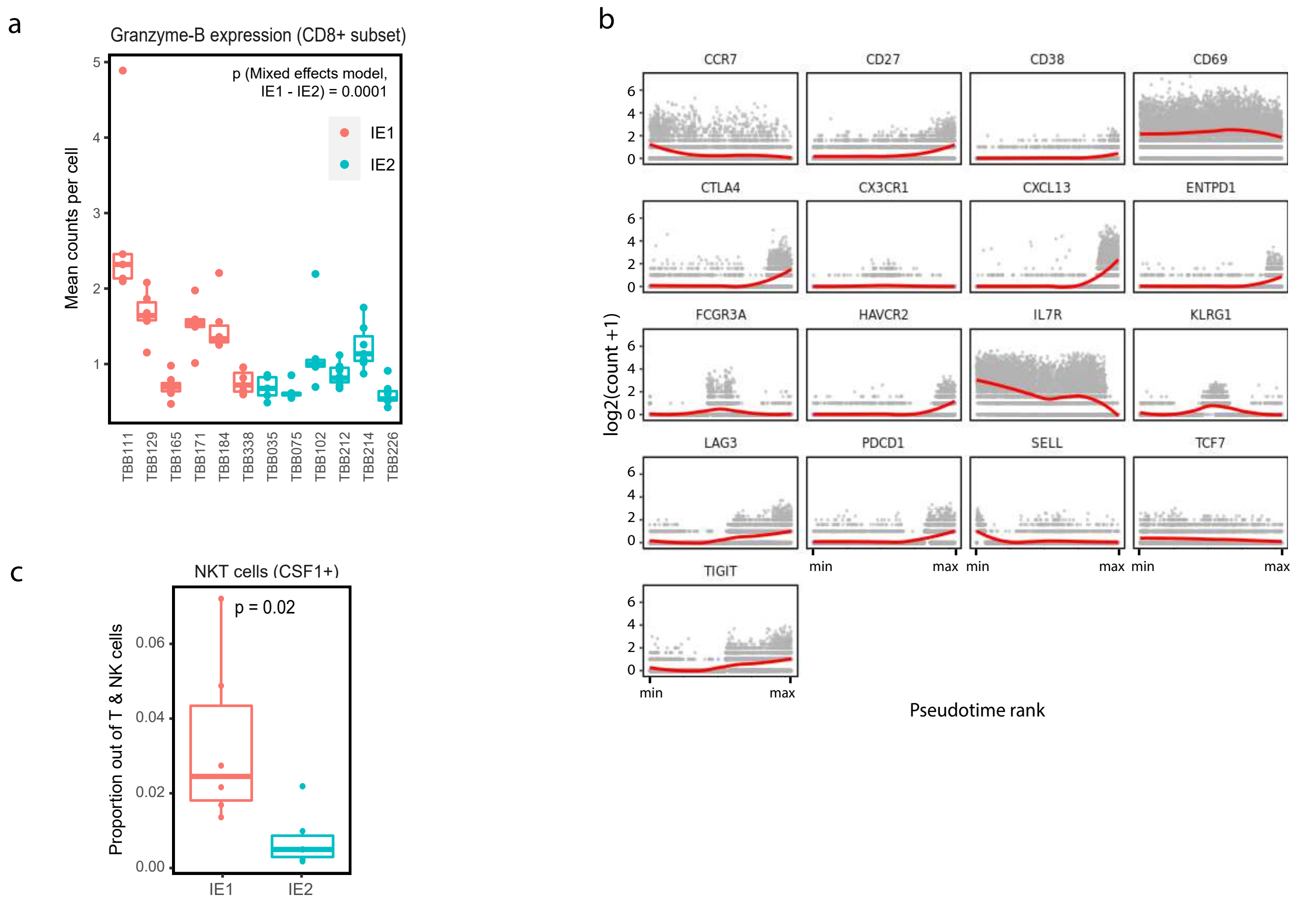

Supplementary Fig. 9: Marker genes associated with exhaustion over pseudotime and IMC validation of exhaustion-related cytotoxic signatures. (a) Boxplot comparing the mean Granzyme-B expression in CD8+ T cells between IE2 and samples based on IMC data (Protein panel). Each dot represents one image (n total = 77 images). A mixed effects model was fitted on the sqrt-transformed data. **(b)** Single-cell expression along pseudotime for transcript levels used as input of the Ouija pseudotime computation (GZMB, FASL, TNF, IFNG, and GZMK were also used as input and are included in the main figure). Red line corresponds to locally estimated scatter-plot smoothing (LOESS) curve. n = 11.200 single T cells from 14 patient samples (800 T cells randomly subset for each sample). **(c)** Boxplot of IMC RNA Panel Data comparing the NKT cell proportions in IE1 and IE2 tumors. A two-sided Wilcoxon rank sum test was used for statistical analysis.

For scatterplots, Spearman correlation coefficient (two-tailed test) and p value are indicated. Boxplot centers indicate the group median, boxplot bodies show IQR, and whiskers extend to the largest and the smallest value lying within 1.5 times the IQR above the 75th percentile and below the 25th percentile, respectively.

.

Supplementary Fig.10

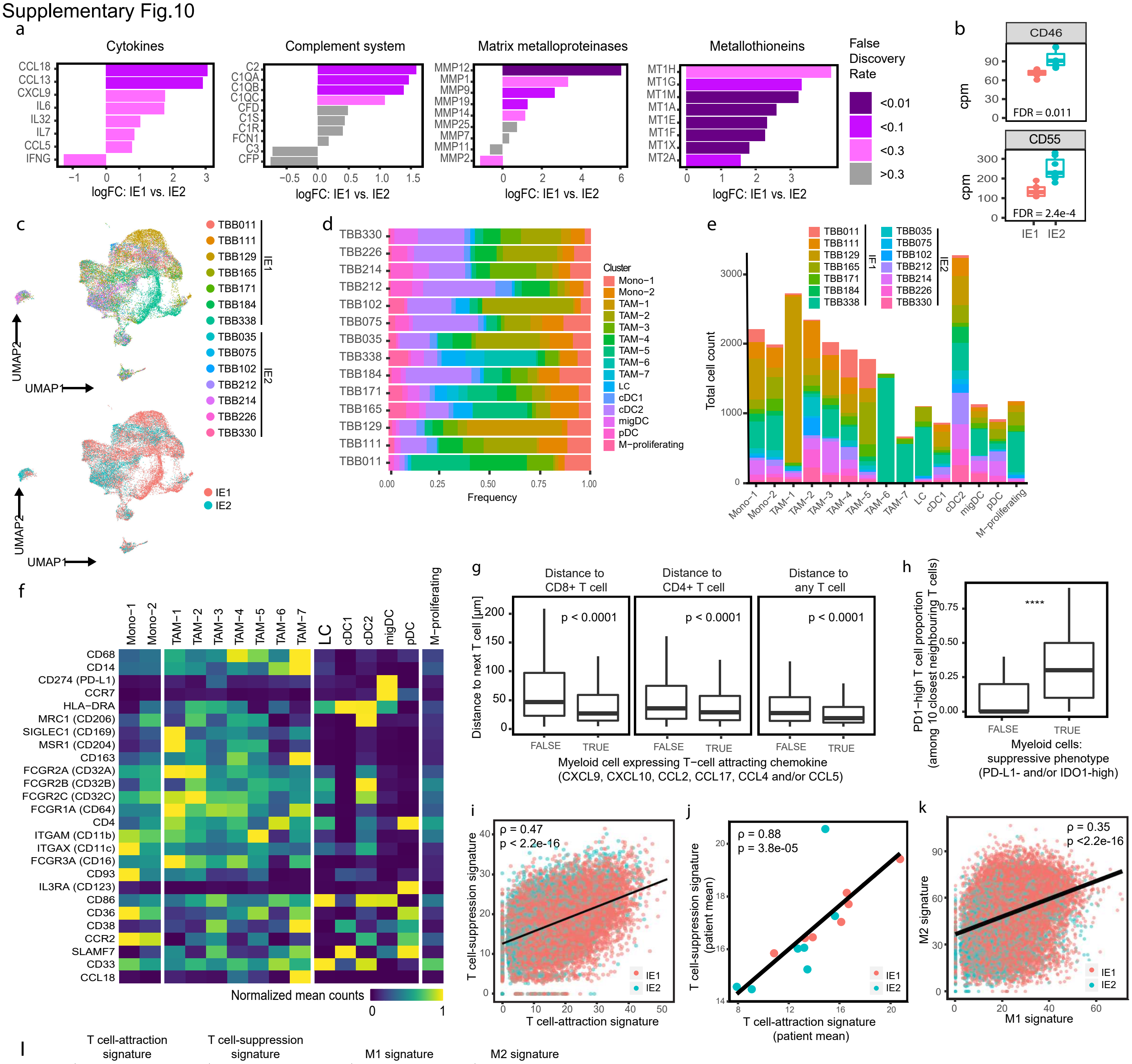

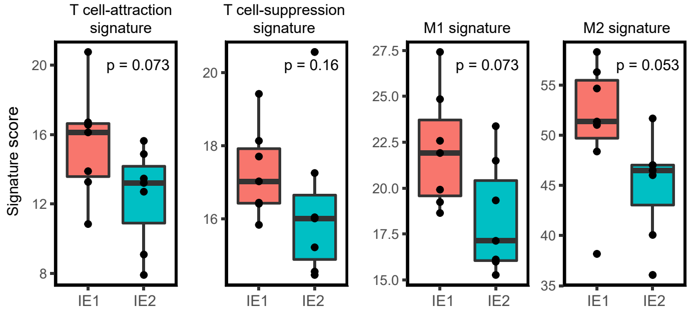

Supplementary Fig. 10: Detailed transcriptomics-based myeloid cell subcluster analysis. (a) Bar plots showing differential expression of the indicated genes between myeloid cells from IE1 and IE2 tumors in pseudobulk patient-averaged scRNA-seq data. The four graphs show levels of transcripts representing the indicated categories. For each category, with the exception of cytokines, all genes with an expression level >1.5 cpm in the higher expressing IE were included. For cytokines, the eight genes with the lowest FDR were included. **(b)** Boxplots comparing the T and NK patient-averaged pseudobulk expression of CD46 and CD55 in IE1 and IE2 samples (n = 14 independent patient samples). **(c)** UMAP of 26,000 myeloid cells colored by patient (left) and IE (right). **(d)** Stacked barplots of relative myeloid cluster frequencies per patient. **(e)** Stacked barplots showing myeloid cluster composition by patient. **(f)** Heatmap showing normalized average expression of myeloid markers commonly used for subtype classification and/or FACS and CyTOF experiments. **(g)** Boxplots comparing distances to the next T cell from myeloid cells that express at least one of the measured T cell-attracting cytokines versus other myeloid cells, based on IMC RNA Panel data. Only non-TLS images were included. **(h)** Boxplots comparing the proportion of PD-1high T cells in the 10 T cells spatially closest to myeloid cells with and without a suppressive phenotype based on IMC Protein panel data. Tregs were excluded for this analysis. **(i)** Single-cell scatterplot of the T cell-attraction score versus the T cell-suppression score. For panels I-K, scores are defined from scRNASeq data. **(j)** Scatterplot of the mean T cell-suppression score versus the mean T cell-attraction score for all patients colored by IE. **(k)** Single-cell scatterplot of the M1 score versus the M2 score. **(l)** Boxplots comparing the mean T cell-attraction, T cell-suppression, M1, and M2 scores for myeloid cells from IE1 and IE2 tumors (n = 14 independent patient samples).

For scatterplots, Spearman correlation coefficient (two-tailed test) and p value are indicated. For boxplots, two-sided Wilcoxon rank sum test was used for statistical analysis. Boxplot centers indicate the group median, boxplot bodies show IQR, and whiskers extend to the largest and the smallest value lying within 1.5 times the IQR above the 75th percentile and below the 25th percentile, respectively.

Supplementary Fig. 11

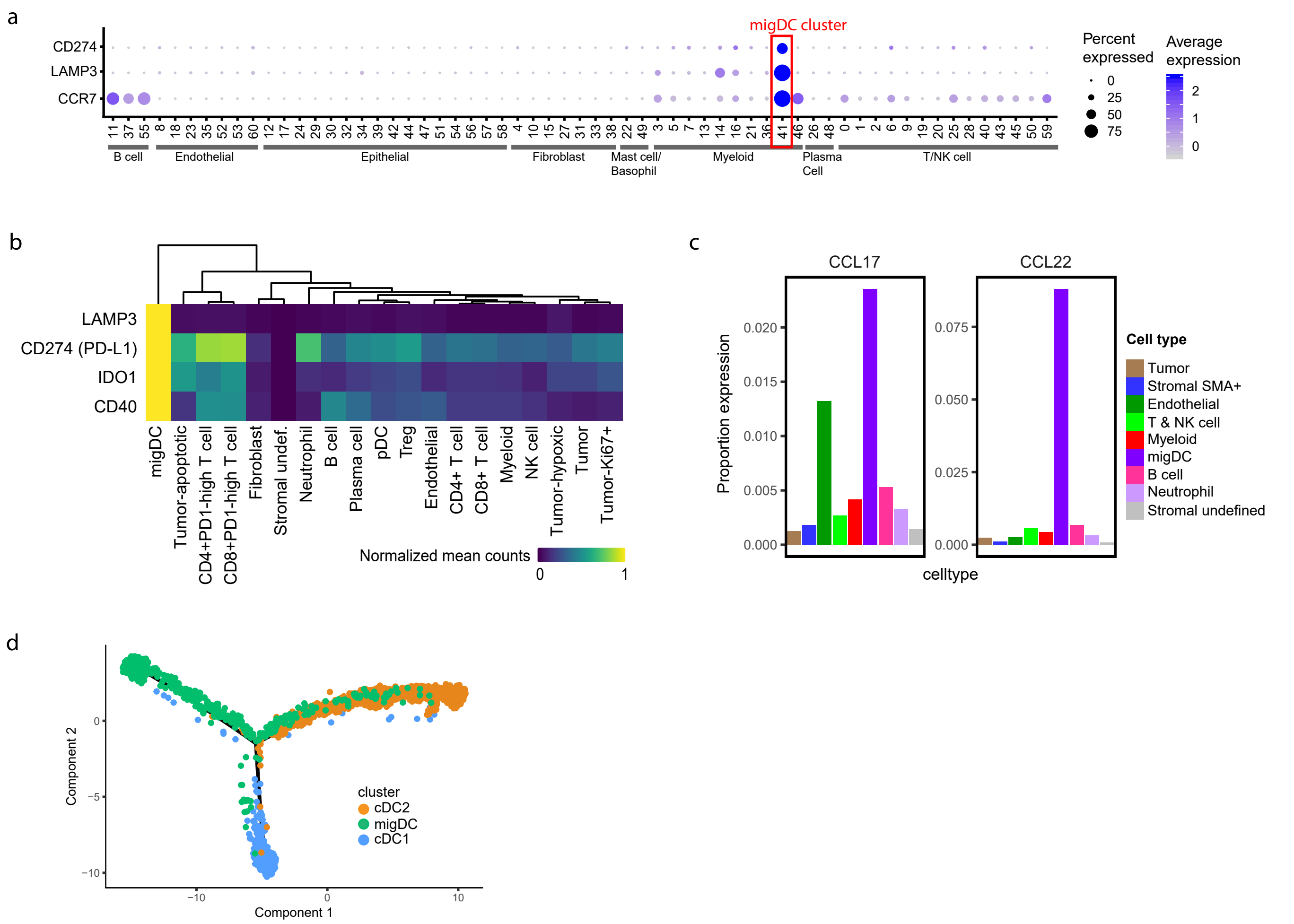

Supplementary Fig. 11: Expression profile and pseudotime developmental trajectory of migDCs. (a) DotPlot of scRNA-seq data showing expression of CD274, LAMP3, and CCR7 across all original high-resolution clusters of the full dataset. **(b)** Heatmap of IMC Protein Panel data showing normalized average expression of LAMP3, PD-L1, IDO, and CD40 proteins for all cell subtypes. **(c)** Bar plots of IMC RNA Panel data displaying percentage of each cell type that expresses CCL17 (left) or CCL22 (right) mRNA. **(d)** Monocle2 trajectory inference of cDC subsets and migDCs based on scRNA-seq data.

Supplementary Fig. 12

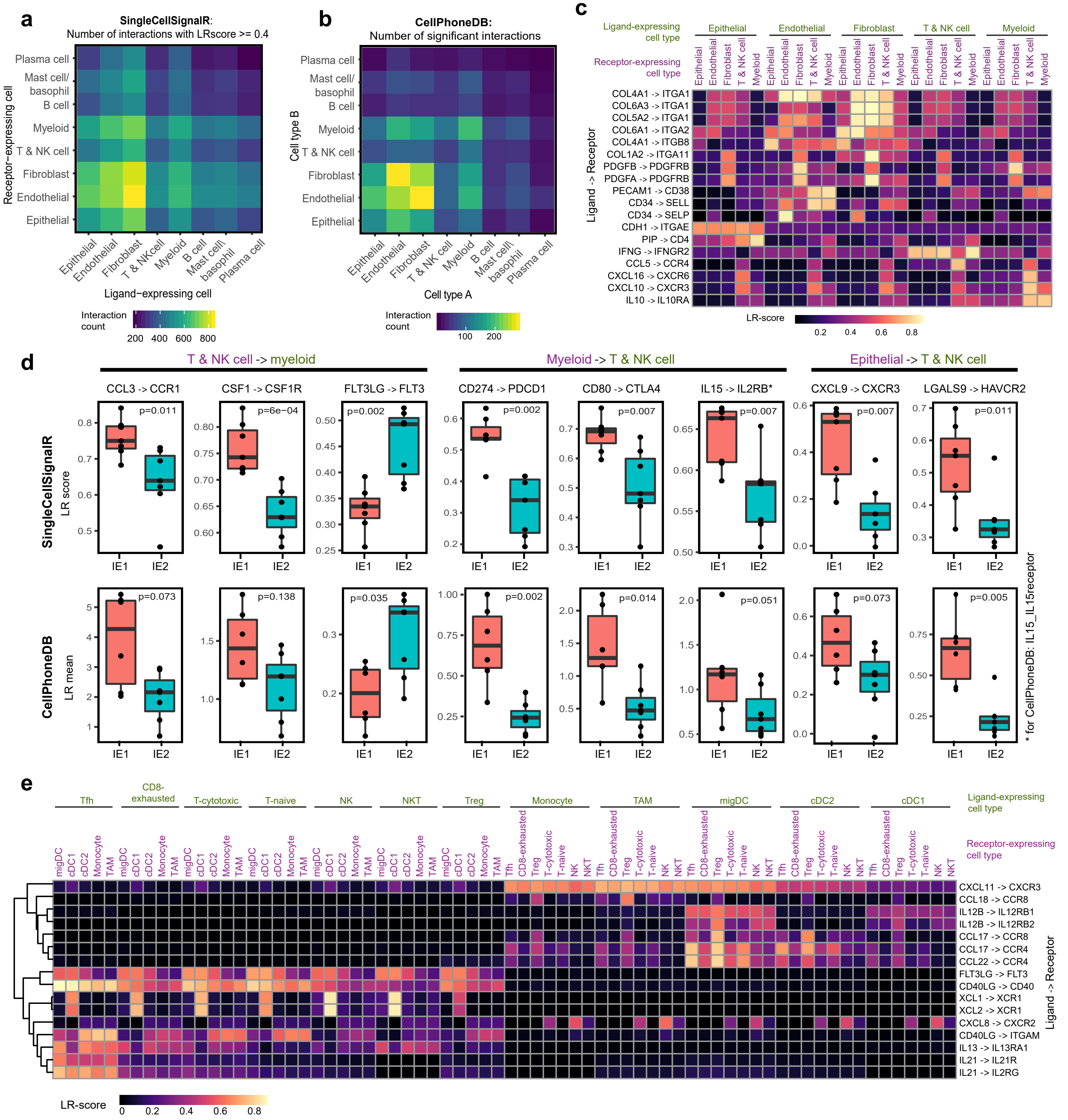

Supplementary Fig. 12: A map of predicted ligand-receptor interactions across exhausted and non-exhausted breast tumor microenviron-ments. (a) Heatmap depicting the number of interactions with an LR score > 0.4 between all main cell type pairs, as given by Single-CellSignalR. **(b)** Heatmap depicting the number of significant interactions between all main cell type pairs as given by CellPhoneDB. The heatmap is symmetric because LR pairs are here not treated as directional. Significance of interactions was calculated by the CellPhoneDB algorithm. **(c)** Heatmap showing LR score for a selection of ligand-receptor interactions over the five most frequent cell type pairs. From the 100 top-scoring ligand-receptor pairs for each cell type pair, the five ligand-receptor pairs with the highest coeffi-cient of variation across cell type pairs were extracted. Ligand-receptor pairs were then selected from this subset based on the strength of literature evidence and biological interpretability. Full SingleCellSignalR results in Supplementary Data 6. **(d)** Boxplots comparing LR scores (from SingleCellSignalR, top) and LR means (from CellPhoneDB, bottom) for a selection of ligand-receptor pairs in IE1 and IE2 tumors. Two-sided Wilcoxon rank sum test was used for statistical analysis. Variations in p values can be explained by the different interaction scoring approach employed by the two methods. Boxplot centers indicate the group median, boxplot bodies show IQR, and whiskers extend to the largest and the smallest value lying within 1.5 times the IQR above the 75th percentile and below the 25th percentile, respectively. n = 14 biologically independent patient samples. **(e)** Heatmap showing the LR scores for a selection of LR interactions over the main T and NK-myeloid metacluster pairs. From the 100 top-scoring LR pairs for each metacluster pair, the five LR pairs with the highest coefficients of variation across metacluster pairs were extracted. LR pairs were then selected from this subset based on the strength of literature evidence and biological interpretability (full SingleCellSignalR resultsin Supplementary Data 6). Less relevant cell types (LC, pDC, T-mixed) as well as myeloid-myeloid and T and NK autopairs were excluded for clarity of the figure. M indicates myeloid metacluster, T indicates T and NK cell metacluster.

Supplementary Fig. 13

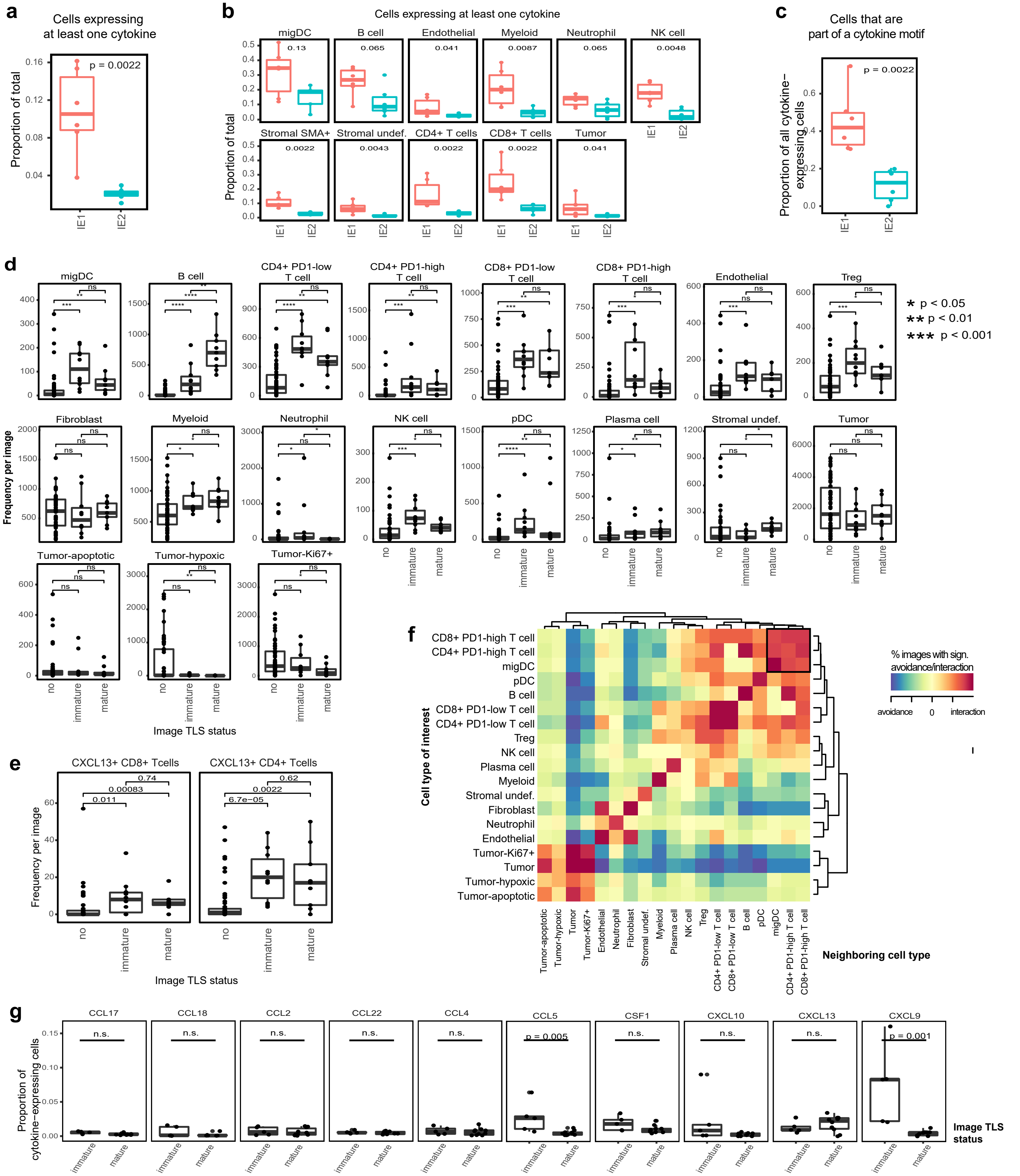

Supplementary Fig. 13: IMC analysis confirms elevated cytokine expression in IE1 and reveals TLS-specific cell type distribution. (a) Boxplot comparing the proportion of cytokine-expressing cells for IE1 and IE2 samples. Each dot represents a sample. Only non-TLS images were included. **(b)** Boxplot compar-ing the proportion of cytokine-expressing cells for IE1 and IE2 samples separated by cell type. **(c)** Boxplots comparing the proportions of cytokine-express-ing cells that are part of a cytokine motif for IE1 and IE2 samples. Only non-TLS images were included. For panel A-C: n = 12 biologically independent patient samples. **(d)** Boxplots comparing the frequency of all cell subtypes for non-TLS, immature TLS, and mature TLS images. The analysis is based on the Protein Panel data. **(e)** Boxplots comparing the frequency of CXCL13+/CD8+ and CXCL13+/CD4+ T cells for non-TLS, immature TLS, and mature TLS images. **(f)** Heat map indicating significant pairwise cell type interaction or avoidance summarized across the two-sided permutation tests on all TLS images (mature and immature) of the Protein Panel dataset (n = 19 images, 1,000 permutations each). Square color indicates the percentage of images with a significant cell-cell interaction or avoidance ($p < 0.01$). **(g)** Boxplots comparing the proportion of cells expressing each individual cytokine in images containing immature TLS regions and images containing mature TLS regions.
For panel D, E and G: n = 77 images from a total of 12 patients.
For boxplots, two-sided Wilcoxon rank sum test was used for statistical analysis. Boxplot centers indicate the group median, boxplot bodies show IQR, and whiskers extend to the largest and the smallest value lying within 1.5 times the IQR above the 75th percentile and below the 25th percentile, respectively.

Supplementary Fig. 14

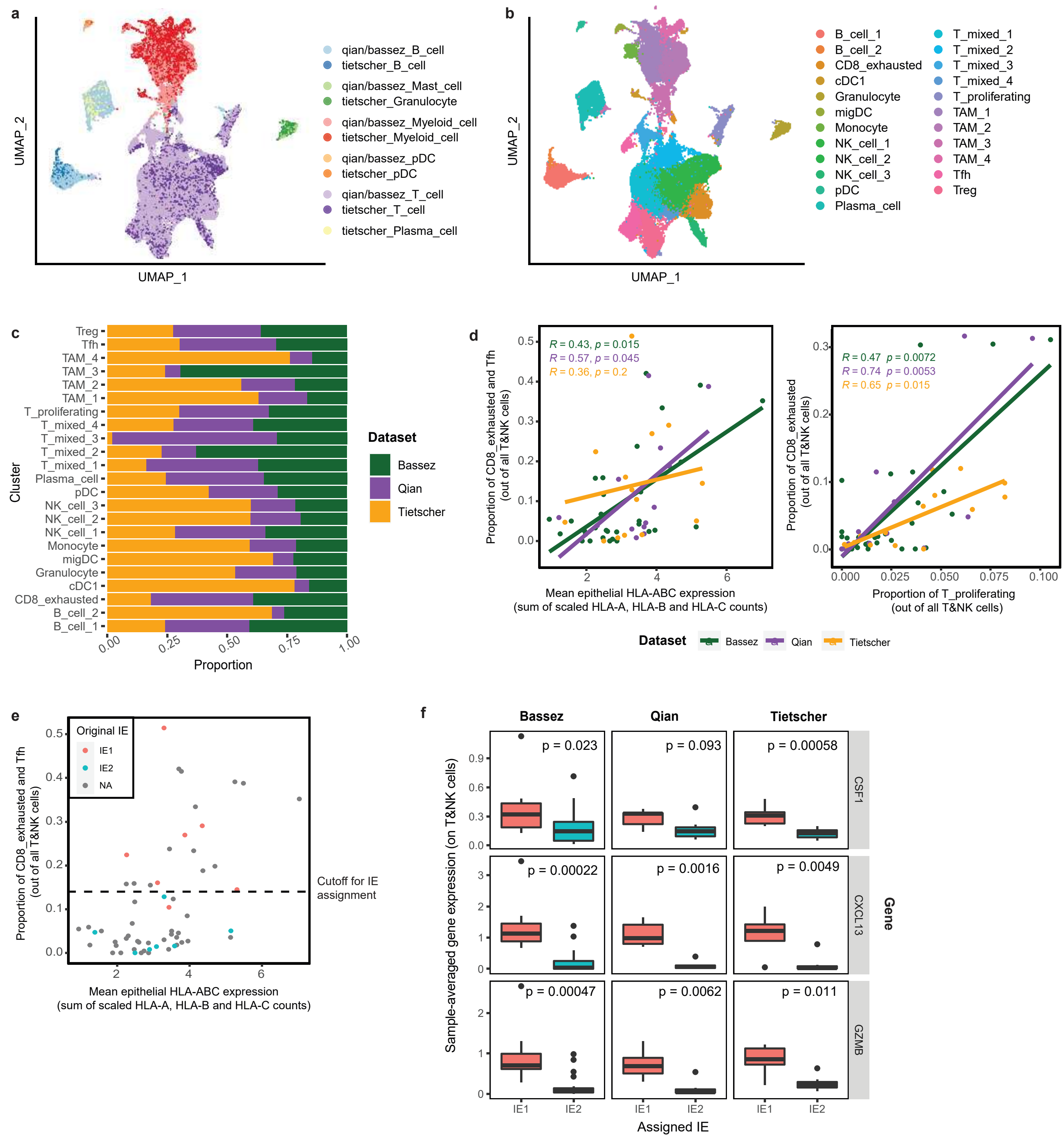

Supplementary Fig. 14: Similar patterns of immune exhaustion are observed across published datasets. (a) UMAP of merged Bassez (1), Qian (2), and Tietscher datasets, colored by original cell type annotation and dataset. Only immune cells were included and 20.000 cells per dataset were randomly subset. The Bassez and Qian datasets were previously published (1, 2). **(b)** Same UMAP as in A, colored by newly generated and annotated clusters. **(c)** Stacked barplot showing the proportion of cells from each dataset in each cluster. **(d)** Left: Scatterplot of the proportion of CD8_exhausted and Tfh cells (out of all T&NK cells) versus the mean epithelial HLA-ABC expression for each patient sample, colored by dataset. Right: Scatterplot of the proportion of CD8_exhausted (out of all T&NK cells) versus the proportion of T_proliferating (out of all T&NK cells) for each patient sample, colored by dataset. Spearman correlation coefficient and p value are indicated for each dataset separately (two-tailed test). **(e)** Same scatterplot as in D (left), colored by original IE (Tietscher dataset). The dashed line indicates the cutoff that was selected to assign IE class to the Bassez (1) and Qian (2) samples. **(f)** Comparison of the CSF1, CXCL13 and GZMB expression levels of T&NK cells from samples that were assigned to IE1 versus samples assigned to IE2, separated by dataset. For the Tietscher dataset, the original IE class was used. Two-sided Wilcoxon rank sum test was used to calculate p-values. Boxplot centers indicate the group median, boxplot bodies show IQR, and whiskers extend to the largest and the smallest value lying within 1.5 times the IQR above the 75th percentile and below the 25th percentile, respectively. n = 31 patient samples from Bassez dataset (1), 13 patient samples from Qian dataset (2), 14 patient samples from Tietscher (this work).

NCOMMS-21-50604B Supplementary References

1. Bassez, A. *et al.* A single-cell map of intratumoral changes during anti-PD1 treatment of patients with breast cancer. *Nature Medicine* (Springer US, 2021). doi:10.1038/s41591-021-01323-8.
2. Qian, J. *et al.* A pan-cancer blueprint of the heterogeneous tumor microenvironment revealed by single-cell profiling. *Cell Res.* **30**, 745–762 (2020).
