# Supplementary material for: A comprehensive single-cell map of T cell exhaustion-associated immune environments in human breast cancer
Source: Nat Commun. 2023 Jan 6;14:98. doi: 10.1038/s41467-022-35238-w (PMC9822999; doi:10.1038/s41467-022-35238-w)
Supplement: Supplementary file 3 — Description of Additional Supplementary Files [file 41467_2022_35238_MOESM3_ESM.pdf]

**Description of Additional Supplementary Files**

File Name: Supplementary  Data 1
Description: Clinical Data.

File Name: Supplementary Data 2
Description: scRNASeq_Data

File Name: Supplementary Data 3
Description: Cluster annotation data.

File Name: Supplementary Data 4
Description: Antibody panel information.

File Name: Supplementary  Data 5
Description: Results of pseudobulk analyses.

File Name: Supplementary Data 6
Description: Gene lists for signatures.

File Name: Supplementary Data 7
Description: SingleCellSignalR output data.

File Name: Supplementary Data 8
Description: CellPhoneDB output data.

File Name: Supplementary Data 9
Description: IMC ROI information.
