# Supplementary material for: A comprehensive single-cell map of T cell exhaustion-associated immune environments in human breast cancer
Source: Nat Commun. 2023 Jan 6;14:98. doi: 10.1038/s41467-022-35238-w (PMC9822999; doi:10.1038/s41467-022-35238-w)
Supplement: Supplementary file 13 — Reporting Summary [file 41467_2022_35238_MOESM13_ESM.pdf]

nature portfolio

Corresponding author(s): Bernd Bodenmiller

Last updated by author(s): Nov 16, 2022

Reporting Summary

Nature Portfolio wishes to improve the reproducibility of the work that we publish. This form provides structure for consistency and transparency in reporting. For further information on Nature Portfolio policies, see our Editorial Policies and the Editorial Policy Checklist.

Statistics

For all statistical analyses, confirm that the following items are present in the figure legend, table legend, main text, or Methods section.

n/a	Confirmed	
☐	☒	The exact sample size (*n*) for each experimental group/condition, given as a discrete number and unit of measurement
☐	☒	A statement on whether measurements were taken from distinct samples or whether the same sample was measured repeatedly
☐	☒	The statistical test(s) used AND whether they are one- or two-sided<br>*Only common tests should be described solely by name; describe more complex techniques in the Methods section.*
☐	☒	A description of all covariates tested
☐	☒	A description of any assumptions or corrections, such as tests of normality and adjustment for multiple comparisons
☐	☒	A full description of the statistical parameters including central tendency (e.g. means) or other basic estimates (e.g. regression coefficient) AND variation (e.g. standard deviation) or associated estimates of uncertainty (e.g. confidence intervals)
☐	☒	For null hypothesis testing, the test statistic (e.g. *F*, *t*, *r*) with confidence intervals, effect sizes, degrees of freedom and *P* value noted<br>*Give P values as exact values whenever suitable.*
☒	☐	For Bayesian analysis, information on the choice of priors and Markov chain Monte Carlo settings
☒	☐	For hierarchical and complex designs, identification of the appropriate level for tests and full reporting of outcomes
☐	☒	Estimates of effect sizes (e.g. Cohen's *d*, Pearson's *r*), indicating how they were calculated

Our web collection on statistics for biologists contains articles on many of the points above.

Software and code

Policy information about availability of computer code

Data collection	Only instrument-internal software was used for data collection (Fluidigm CyTOF Software v7.0). Details about the acquisition instruments can be found in the Methods section.
Data analysis	For pre-processing of scRNA-seq data, the CellRanger pipeline (10x Genomics, v3.0.1) was used. Downstream analysis was performed in R v.3.6.1 with the exception of ligand-receptor analysis using CellPhoneDB package, which was performed in Python (v3.7). All major R packages used are listed in the Methods section and include DoubletFinder (v2.0), Seurat (v3.0.2), EdgeR (v3.26.5), ouija (v0.99.1), Slingshot (v1.7.3), Monocle (v2.12) and SingleCellSignalR (v1.6.0). Pre-processing of Imaging Mass Cytometry data was performed using ilastik (v1.3.3) and CellProfiler (v3.1.9) and downstream analysis was performed in R (v4.0.2). Major R packages used for IMC downstream analysis include SingleCellExperiment (v1.10.1), scater (v1.16.2), neighbouRhood (v0.4) and cytomapper (v1.0.0). For the visualization and analysis of whole slide IF images, Zeiss Axioscan Z.1 software was used. All custom analysis code has been deposited in the following repositories.<br>scRNA-Seq data analysis: https://github.com/BodenmillerGroup/BCexh_scRNAseq<br>IMC data analysis: https://github.com/BodenmillerGroup/BCexh_IMC<br>Integration with public datasets: https://github.com/BodenmillerGroup/BC_RNAseq_integration

For manuscripts utilizing custom algorithms or software that are central to the research but not yet described in published literature, software must be made available to editors and reviewers. We strongly encourage code deposition in a community repository (e.g. GitHub). See the Nature Portfolio guidelines for submitting code & software for further information.

Data

Policy information about availability of data

All manuscripts must include a data availability statement. This statement should provide the following information, where applicable:

- Accession codes, unique identifiers, or web links for publicly available datasets
- A description of any restrictions on data availability
- For clinical datasets or third party data, please ensure that the statement adheres to our policy

RNA-seq data have been deposited in the ArrayExpress database at EMBL-EBI (www.ebi.ac.uk/arrayexpress) under accession number E-MTAB-10607. Imaging Mass Cytometry data have been deposited on Zenodo (DOI: 10.5281/zenodo.4911135). The read count data and metadata from the Bassez 2021 dataset was downloaded from https://lambrechtslab.sites.vib.be/en/single-cell. The counts matrix and metadata from the Qian 2020 breast cancer dataset was downloaded from https://lambrechtslab.sites.vib.be/en/pan-cancer-blueprint-tumour-microenvironment.

Field-specific reporting

Please select the one below that is the best fit for your research. If you are not sure, read the appropriate sections before making your selection.

☒ Life sciences ☐ Behavioural & social sciences ☐ Ecological, evolutionary & environmental sciences

For a reference copy of the document with all sections, see nature.com/documents/nr-reporting-summary-flat.pdf

Life sciences study design

All studies must disclose on these points even when the disclosure is negative.

Sample size	For this study, 14 patient samples (7 for each of the investigated immune environments) were sequenced and FFPE blocks for 12 of these samples (6 of each group) were additionally stained and imaged with IMC Sample size was limited by the number of samples previously characterized by mass cytometry (to determine immune environment state) for which a sufficient number of viable cells was available. For IF imaging and CD8+ T cell infiltration assessment, the 12 samples imaged by IMC plus another 13 samples that had been previously characterized by mass cytometry (Wagner et al, 2019) were used (total n=25).
Data exclusions	No data was excluded from this study. All samples that were successfully sequenced and/or stained and imaged were used in our data analysis.
Replication	All cell types, clusters and interactions were identified in multiple patient samples, and major findings from RNA-seq were confirmed on a protein-level by IMC imaging.
Randomization	Patients were selected to be equally distributed between the two immune microenvironments. RNA sequencing runs were performed in 7 batches and each batch contained one sample of each biological group to minimize the overlap between technical and biological variation. IMC image acquisition order was random.
Blinding	For IMC, all samples were stained simultaneously; selection of ROIs and TLS scoring was blinded to clinical data. For RNA-seq analysis, blinding was not relevant because only quantitative (no qualitative or subjective) measures were used.

Reporting for specific materials, systems and methods

We require information from authors about some types of materials, experimental systems and methods used in many studies. Here, indicate whether each material, system or method listed is relevant to your study. If you are not sure if a list item applies to your research, read the appropriate section before selecting a response.

Materials & experimental systems

n/a	Involved in the study	
☐	☒	Antibodies
☒	☐	Eukaryotic cell lines
☒	☐	Palaeontology and archaeology
☒	☐	Animals and other organisms
☐	☒	Human research participants
☒	☐	Clinical data
☒	☐	Dual use research of concern

Methods

n/a	Involved in the study	
☒	☐	ChIP-seq
☒	☐	Flow cytometry
☒	☐	MRI-based neuroimaging

Antibodies

Antibodies used	All Antibody information is available in Supplementary Data 4

Validation	All antibodies were validated by immunofluorescence prior to isotope-polymer conjugation, and by IMC after conjugation. Antibody validation included staining of known marker-positive and marker-negative tissue types (including tonsil, lymph node, spleen and different tumor types), assessment of staining pattern across the tissue compared to staining patterns published in the Human Protein Atlas (proteinatlas.org), cell type specificity (assessed by co-staining with other markers) and intracellular location.

Human research participants

Policy information about studies involving human research participants

Population characteristics	All patient data is available in Supplementary Data 1. Histopathological data was obtained from the individual pathology reports.
Recruitment	The specimens derived from patients diagnosed with primary breast cancer between 2015 and 2018 at the breast cancer centers at St. Johannes Hospital Dortmund and Institute of Pathology at Josefshaus (Germany) and the University Hospital Giessen and Marburg, Marburg site (Germany) . All specimens were collected in collaboration with the Patient's Tumor Bank of Hope (PATH, Germany).
Ethics oversight	Tissue and health-related data were collected under approval of the Cantonal Ethics Commmittee Zurich (Kantonale Ethikkommission Zürich, #2016-00215) and the faculty of medicine ethics committee at Friedrich-Wilhelms-University Bonn (#255/06).

Note that full information on the approval of the study protocol must also be provided in the manuscript.
